# Loneliness and its associations with oral and general health and socio-demographic factors in 80- and 90-year-old Swedes

**DOI:** 10.1186/s12903-025-07293-4

**Published:** 2025-11-22

**Authors:** Anders Johansson, Ridwaan Omar, Stein Atle Lie, Josefin Sannevik, Berit Mastrovito, Ann-Katrin Johansson

**Affiliations:** 1 Department of Clinical Dentistry— Prosthodontics, Faculty of Medicine, University of Bergen, Årstadvn. 19, Bergen, 5009 Norway; 2 Department of Thoracic Medicine, Haukeland University Hospital, Bergen, Norway; 3 College of Dentistry, Kuwait University, Safat, Kuwait; 4 Center for Translational Oral Research (TOR), Department of Clinical Dentistry, Faculty of Medicine, University of Bergen, Bergen, Norway; 5 Department of Dentistry, Örebro County Council, Örebro, Sweden; 6 Dental Commissioning Unit, Östergötland County Council, Linköping, Sweden; 7 Department of Clinical Dentistry – Cariology, Faculty of Medicine, University of Bergen, Bergen, Norway

**Keywords:** Aged, 80 and over, Dental health surveys, Geriatrics, Social isolation

## Abstract

**Background:**

Loneliness has a negative impact on well-being and is affected by a multitude of different variables, among these being oral health status. This study assesses tentative associations between perceived loneliness and oral and general health and socio-demographic factors in elderly Swedes.

**Methods:**

In this cross-sectional study, all 80- and 90-year-olds living in two counties in southern Sweden, were sent a questionnaire in 2022. The questionnaire comprised questions on socio-demographic-, general- and oral health-related status. A question on loneliness was included as: *“Did you experience more loneliness during the Corona pandemic?”* (with alternatives: 1 = ”yes, always”, 2 = ”often”, 3 = ”seldom”, and 4 = ”never”). Descriptive analyses and binary multivariable logistic regression analyses both totally and stratified by sex were performed to study the associations between self-reported loneliness and health and socio-demographic factors.

**Results:**

The total population comprised 8203 individuals (4541 women, 3662 men) out of which 5202 responded (2783 women and 2419 men) giving an overall response rate of 63%. Overall, 34.3% of 90-year-olds reported often or always loneliness compared to 26.9% of 80-year-olds (*p* < 0.001). Women reported significantly more loneliness than men both in 90-year-olds (40.2% vs. 25.3%; *p* < 0.001) and in 80-year-olds (32.4% vs. 21.1%; *p* < 0.001). Loneliness among women was significantly higher in 90-year-olds than 80-year-olds (40.2% vs. 32.4%; *p* = 0.005), but not so among men (25.3% vs. 21.1%; *p* = 0.23). Women who were unmarried and perceived they had impaired general health were significantly associated with reported loneliness. Regarding oral parameters, lesser belief about keeping their teeth for their remaining life, night-time xerostomia, taste changes, bleeding gums, bad breath and impact from Oral Impact on Daily Performance (OIDP) were all significantly associated with loneliness. Stratified by sex, the corresponding findings for women were night-time xerostomia, bleeding gums and bad breath, whereas for men, only taste changes and impact from OIDP were associated with loneliness.

**Conclusions:**

Several oral health-related variables in addition to multiple sociodemographic and health factors are associated with self-reported loneliness. 90-year-olds reported greater loneliness than 80-year-olds and women were more affected by loneliness than men. In addition, women had more oral health-related variables associated with loneliness compared to men.

**Supplementary Information:**

The online version contains supplementary material available at 10.1186/s12903-025-07293-4.

## Introduction

Loneliness, the subjective negative feeling of being lonely, has lately been in focus, particularly among elderly people, and in connection with the COVID-19 pandemic. The WHO Commission on Social Connection (2024–2026) addresses the negative consequences of loneliness and social isolation and calls for strategies and solutions to counteract its negative consequences on peoples’ health and overall well-being [1]. Globally, it has been estimated that 25% of the elderly experience social isolation. Furthermore, according to research, loneliness has a comparable effect on mortality as do other well-documented risk factors such as smoking, obesity, and physical inactivity [2]. Accordingly, the WHO has advocated a strategy for reducing social isolation and loneliness among elderly people that includes implementation of effective interventions based on evidence-based research as well as to enhance its political priority [3].

### Prevalence of loneliness and consequences from the COVID-19 pandemic

A systematic review covering 120 000 elderly people from 29 high income countries, mainly European, in the period 2008–2020, found that the pooled estimate of self-reported loneliness was 28.5%. For individuals 65–75 and over 75 years of age, the prevalence was 27.6% and 31.3%, respectively [4]. In a Swedish national survey, conducted at the end of the COVID-19 pandemic (December 2022), of individuals aged 85 years or older, about 40% of women and close to 30% of men reported loneliness and isolation [5]. In addition, loneliness varies geographically and has, for example, been found to be less common in the northern part of Europe than in the eastern and southern parts [4].

A repeated cross-sectional Swedish study covering the years 1992–2014, among individuals ≥ 77 years did not, in contrast to what many believed, show any increase in the prevalence of loneliness during the 12-year study period. However, social- and health-related variables showed a stronger association with loneliness than socio-demographic variables [6].

A later study, investigating the effect of the COVID-19 pandemic, found an increase in self-reported loneliness compared to before the pandemic, albeit with a small effect size [7]. This is analogous with the findings of an Austrian sample of community-dwelling elderly, aged 60 and above, where reported loneliness was only slightly higher during the pandemic compared to before. Interestingly, those who lived alone did not report more loneliness during this period whereas those who lived with someone did [8]. As regards differences for marital status, a study covering 26 European countries and Israel found that short-term widowed men and short- and medium-term widowed and divorced women were at greater risk for increased loneliness reported during the COVID-19 pandemic [9].

### Loneliness and oral health

In a large study of 26,168 participants aged 50 and above, social isolation and loneliness were found to be associated with poor oral health and cognitive status [10]. Complete loss of natural teeth and reduced oral health-related quality of life are other consequences that have been associated with loneliness [11–13]. In a systematic review, both objective and perceived social isolation, with a moderate to strong level of evidence, was found to be associated with worse oral health in community-dwelling older adults aged 60 years or older, while the frequency of social contacts in terms of meeting friends had an inconsistent association with oral health [14].

### Associations between loneliness, social- and general health-related factors

An extensive systematic review of longitudinal studies reported on risk factors for loneliness in individuals over 60 years of age. Although 120 unique risk factors were studied, those who showed consistent associations with loneliness were related to living alone/had suffered loss of a partner, impaired social network/activity and health related parameters such as poor self-perceived health and depression [15]. Other consequences of loneliness/social isolation in the elderly include increased risk for cardiovascular disease, suicidal ideation (aged 55 +), reduced physical activity and impaired quality of life [16–19]. The world’s populations are steadily becoming both larger and older which will lead to an increased number of elderly feeling lonely. There are few reports targeting the very old in regard to loneliness and more needs to be known on its effect on health and well-being in this specific age group.

The aims of this study were to examine associations between perceived loneliness reported during the COVID-19 pandemic in 2022, and oral and general health and socio-demographic factors in 80- and 90-year-old Swedes with special focus on differences between women and men.

## Material and methods

All individuals who lived in two counties in southern Sweden in 2022, and were born in 1932 (*n* = 1904, 90 years old) and in 1942 (*n* = 6299, 80 years old) were sent a questionnaire by regular mail in May 2022. A reminder was posted after 6 weeks to those who did not answer. All addresses were obtained from Statistics Sweden which is a government agency that produces official statistics.

This cross-sectional investigation was part of a prospective longitudinal survey that started in 1992 for the 1942 cohort and in 2007 for the 1932 cohort, and repeated for the respective cohorts every 5th year [20, 21]. All surveys used the same questionnaire with some minor modifications over the years in order to adapt to changing circumstances related to ageing and prevailing conditions, notably the COVID-19 pandemic (Supplementary file “Questionnaire_2022_eng”). The questionnaire comprised questions on socio-demographic-, general-, and oral health-related status. Oral Impact on Daily Performance (OIDP) was included in the survey from 2007 and subsequent surveys. OIDP is a commonly used instrument that measures oral health-related quality of life and has been validated in Swedish [22]. In the 2022 survey an additional question on loneliness was added: *“Did you experience more loneliness during the Corona pandemic?”* with the response alternatives 1 = ”yes*, always*”, 2 = ”*often*”, 3 = ”*seldom*”, and 4 = ”*never*”.

### Statistical analysis

Descriptive analyses on self-reported loneliness stratified by age and sex were executed. Cohort and sex differences in reported loneliness were analyzed with Mann–Whitney U-test. To investigate potential associations (collinearity) between the independent variable, cross-tables and chi-square tests were performed for all combinations of these variables. Associations between the dichotomous dependent variables and the independent variables were analyzed using Poisson regression models with robust variance estimates. The results from these analyses are reported as relative risks (RRs). Initially, an unadjusted Poisson regression analysis was performed between questionnaire-reported tentative associated factors and loneliness. Loneliness during the COVID-19 pandemic was the binary dependent variable and dichotomized as 0 = *“seldom or never”* and 1 = *“yes, always”* or “*often*”. The initial analyses were followed by a sequential approach where the general and oral health-related variables were included as independent variables in the analyses. In the first step in a sequential analysis, all oral health variables were entered into the regression (Model 1) followed by the addition of general health variables in the second step (Model 2). In the final step demographic variables were also included (Model 3). The reason for the sequential approach was to study how additional adjustment possibly altered the association between the oral independent variables and the dependent variables. Hence, in the second step, general health variables, which are assumed to be related to, that is, to explain, the oral health variables, were entered. For the last analyses, demographic variables, assumed to be associated with both the oral and general health variables were entered. The adjusted analyses were performed for all individuals, and in addition for women and men separately (i.e. stratified by sex). Multiple imputation (MI, using logistic regression approach, since all variables were dichotomous) was applied with 50 replications, in order to recreate missing data. The MI approach for missingness of the data will account for missing at random (MAR), in which the missingness can be explained by the variables available for the analyses. *P*-values < 0.05 were considered statistically significant. All analyses were performed using the statistical package Stata 18 (StataCorp LLC, College Station, TX, USA).

## Results

The total population of 80- and 90-year-olds living in the two counties in 2022 was 8203 people, comprising 4541 women (55%) and 3662 men (45%). The overall response rate was 66% (5375/8203). The total population of 90-year-olds (1932 cohort) was 1904 (64% women, 36% men) with a response rate of 54% (*n* = 1032/1904, 60.5% women, 39.5% men). The corresponding figures for the 80-year-olds (1942 cohort) was 6299 (53% women, 47% men) and the response rate was 69% (*n* = 4343/6299, 52% women, 48% men).

**Fig. 1:**
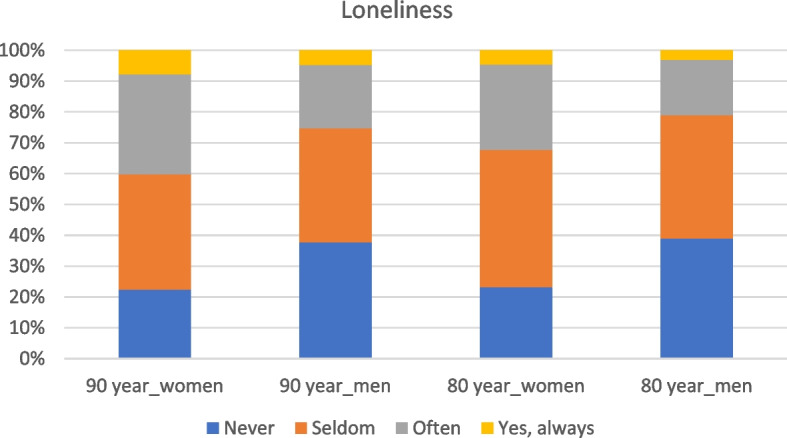
Frequency distribution of reported loneliness among 90-year-olds (1932 cohort, *n* = 968; women = 584; men = 384) and 80-year-olds (1942 cohort, n = 4234; women = 2199; men = 2035) answering the question: *“Did you experience a greater loneliness during the Corona pandemic?”* with the response alternatives; never, seldom, often or yes, always

The number of responses to the question *“Did you experience more loneliness during the Corona pandemic?”* was 5202 corresponding to a response rate of 63% (2783 women and 2419 men) which constitutes the base data for the analyses. Of the respondents, 3518 provided complete data, while 1684 were missing 1 or more variables (Supplementary file Table S1). Frequency distribution of reported loneliness is shown in Fig. 1. Overall, 34.3% reported often or always loneliness among the 90-year-olds compared to 26.9% for the 80-year-olds (*p* < 0.001). Women reported significantly more loneliness compared to men both in 90-year-olds (40.2% vs. 25.3%; *p* < 0.001) and in 80-year-olds (32.4% vs. 21.1%; *p* < 0.001). Self-reported loneliness among women was significantly higher in 90-year-olds compared to the 80-year-olds (40.2% vs. 32.4%; *p* = 0.005), but not so among men (25.3% vs. 21.1%; *p* = 0.23).

### Regression analyses

The independent variables included in the unadjusted regression analysis, with the dichotomized loneliness variable as dependent, were divided into three domains viz. oral health, general health and socio-demographic as shown in Table 1. For the analysis of associations between all the independent variables, included in the models, only 7 showed no statistical association. These were Teeth For Life vs. Year Birth, Xerostomia Night vs. Alcohol and Marital Status, Mouth Opening vs. Year Birth, Bleeding Gums vs. Alcohol and Marital Status, Bad Breath vs. Sex, Tooth Sensitivity vs. Year Birth and Marital Status and Sex, and OIDP vs. Sex (all *p* > 0.05).

**Table 1:** Unadjusted binary logistic regression: Dependent variable: 0 = ”seldom/never loneliness” vs. 1 = ”always/often loneliness”. Description of independent variables, their dichotomizations and *P*-values for the unadjusted association with the dependent variable

Independent variables	Dichotomization	P
*Socio-demographic*		
Year of birth	0 = 1932; 1 = 1942	< 0.001
Sex	0 = man; 1 = woman	< 0.001
Birthplace	0 = other country 1 = Sweden	0.818
Residency	0 = countryside; 1 = large or small city	< 0.001
Social contacts	0 = > 5 weekly social contacts; 1 = 0–5	< 0.001
Housing	0 = other; 1 = own living	0.004
Education	0 = higher secondary school, gymnasium, university; 1 = elementary school (6yrs)	0.996
Marital status	0 = unmarried/divorced/widow(er); 1 = married/cohabiting	< 0.001
*General health*		
Perceived full health	0 = no, not especially/absolutely not; 1 = yes, absolutely/by and large	< 0.001
Own health compared to peers	0 = no, worse/much worse; 1 = yes, much better/better/equally good	< 0.001
Doctor visit last 3 months	0 = no; 1 = yes, several/some/one times	< 0.001
Smoking	0 = stopped/never smoked; 1 = smoking daily/occasionally	0.193
Snuff (smokeless tobacco)	0 = stopped/never snuffed; 1 = snuffing daily/occasionally	0.955
Alcohol intake (spirits, wine, strong beer)	0 = monthly or never; 1. weekly or more often	< 0.001
*Oral health*		
Perception of keeping own teeth for remaining life	0 = yes, maybe, no, probably not/absolutely not/don't know; 1 = yes, absolutely	< 0.001
Teeth satisfaction	0 = no, not especially satisfied/absolutely not satisfied; 1 = yes, very satisfied/by and large satisfied	< 0.001
Ability to chew all kind of food	0 = rather good/not so good/bad; 1 = very good	< 0.001
Satisfaction with teeth esthetics	0 = by and large pleased/not especially/absolutely not; 1 = yes, very pleased	< 0.001
Xerostomia daytime	0 = yes, sometimes, no seldom/never; 1 = yes, often	< 0.001
Xerostomia nighttime	0 = yes, sometimes, no seldom/never; 1 = yes, often	< 0.001
Number of own teeth	0 = missing rather many teeth/almost no teeth left/edentulous; 1 = all teeth left/missing a single tooth	< 0.001
*Do you have trouble with:*		
Burning mouth	0 = some/rather much/great troubles; 1 = no trouble	< 0.001
Oral wounds or blisters	0 = some/rather much/great troubles; 1 = no trouble	< 0.001
Taste changes	0 = some/rather much/great troubles; 1 = no trouble	< 0.001
TMJ pain	0 = some/rather much/great troubles; 1 = no trouble	< 0.001
Mouth opening	0 = some/rather much/great troubles; 1 = no trouble	< 0.001
Bleeding gum	0 = some/rather much/great troubles; 1 = no trouble	< 0.001
Bad breath	0 = some/rather much/great troubles; 1 = no trouble	< 0.001
Tooth sensitivity	0 = some/rather much/great troubles; 1 = no trouble	< 0.001
Tooth wear	0 = some/rather much/great troubles; 1 = no trouble	< 0.001
Caries during last year	0 = no; 1 = yes	< 0.001
Perio problem during last year	0 = no; 1 = yes	< 0.001
Oral Impacts on Daily Performance (OIDP)	0 = no impact; OIDP 1 = impact ≥ 1	< 0.001

Model 1 depicts independent oral health variables that were statistically significant in the unadjusted regression analysis. In Model 2 significant general health variables were entered, and in Model 3 significant socio-demographic variables were included (Tables 2, 3 and 4).

**Table 2:** Adjusted logistic regression analysis for both sexes. Dependent variable: seldom/never loneliness (1) vs. always/often loneliness (0)

**Independent variables**	**Model 1**			**Model 2**			**Model 3**		
	**RR**	**95% CI**	**p**	**RR**	**95% CI**	**p**	**RR**	**95% CI**	**p**
Teeth for life, maybe or no	1	Ref		1	Ref		1	Ref	
Teeth for life, yes	0.86	0.77,0.95	0.003	0.89	0.80,0.98	0.020	0.88	0.80,0.97	0.013
Xerostomia day, sometime or no	1	Ref		1	Ref		1	Ref	
Xerostomia day, yes or often	1.16	1.01,1.32	0.032	1.06	0.93,1.21	0.394	1	0.87,1.14	0.970
Xerostomia night, sometimes or no	1	Ref		1	Ref		1	Ref	
Xerostomia night, yes or often	1.16	1.04,1.30	0.010	1.14	1.02,1.28	0.021	1.13	1.01,1.26	0.032
Taste Change, some	1	Ref		1	Ref		1	Ref	
Taste change, no	0.77	0.69,0.86	< 0.001	0.82	0.73,0.92	0.001	0.85	0.76,0.95	0.005
Mouth opening, some	1	Ref		1	Ref		1	Ref	
Mouth Opening, no	0.86	0.76,0.97	0.015	0.89	0.79,1.01	0.075	0.91	0.81,1.03	0.121
Bleeding gum, some	1	Ref		1	Ref		1	Ref	
Bleeding gum, no	0.85	0.77,0.95	0.004	0.85	0.77,0.95	0.003	0.87	0.78,0.96	0.007
Bad breath, some	1	Ref		1	Ref		1	Ref	
Bad breath, no	0.85	0.77,0.94	0.002	0.86	0.78,0.96	0.005	0.86	0.78,0.95	0.003
Tooth sensitivity, some	1	Ref		1	Ref		1	Ref	
Tooth sensitivity, no	1.11	1,1.23	0.060	1.08	0.97,1.20	0.138	1.11	1,1.23	0.051
OIDP = 0	1	Ref		1	Ref		1	Ref	
OIDP ≥ 1	1.23	1.12,1.36	< 0.001	1.20	1.09,1.32	< 0.001	1.18	1.08,1.30	0.001
Full health, no				1	Ref		1	Ref	
Full health, yes				0.71	0.65,0.79	< 0.001	0.73	0.66,0.80	< 0.001
Alcohol monthly or never				1	Ref		1	Ref	
Alcohol weekly or more				0.90	0.82,0.99	0.023	1.03	0.93,1.13	0.603
Year of birth – 1932							1	Ref	
Year of birth – 1942							0.96	0.86,1.06	0.390
Unmarried							1	Ref	
Married							0.69	0.63,0.76	< 0.001
Men							1	Ref	
Women							1.35	1.23,1.49	< 0.001
*N*	5202			5202			5202		

Model 1: “Oral health”, Model 2: “Oral health + General health” and Model 3: “Oral health + General health + Socio-demographic parameters”

*RR* relative risk, *CI* confidence interval, *p* statistical significance (*p*-value)

**Table 3:** Adjusted logistic regression analysis for women. Dependent variable: seldom/never loneliness vs. always/often loneliness

**Independent variables**	**Model 1**			**Model 2**			**Model 3**		
	**RR**	**95% CI**	**p**	**RR**	**95% CI**	**p**	**RR**	**95% CI**	**p**
Teeth for life, maybe or no	1	Ref		1	Ref		1	Ref	
Teeth for life, yes	0.86	0.76,0.97	0.011	0.88	0.78,0.99	0.039	0.89	0.79,1	0.055
Xerostomia day, sometime or no	1	Ref		1	Ref		1	Ref	
Xerostomia day, yes or often	1.14	0.99,1.32	0.069	1.05	0.91,1.22	0.479	1.05	0.91,1.22	0.496
Xerostomia night, sometimes or no	1	Ref		1	Ref		1	Ref	
Xerostomia night, yes or often	1.13	1,1.28	0.055	1.13	0.99,1.28	0.066	1.14	1.01,1.29	0.037
Taste Change, some	1	Ref		1	Ref		1	Ref	
Taste change, no	0.83	0.73,0.95	0.007	0.88	0.77,1.01	0.064	0.89	0.78,1.02	0.101
Mouth opening, some	1	Ref		1	Ref		1	Ref	
Mouth Opening, no	0.85	0.74,0.98	0.023	0.88	0.77,1.01	0.064	0.88	0.76,1	0.058
Bleeding gum, some	1	Ref		1	Ref		1	Ref	
Bleeding gum, no	0.89	0.79,1.01	0.074	0.89	0.79,1.01	0.064	0.88	0.78,1	0.043
Bad breath, some	1	Ref		1	Ref		1	Ref	
Bad breath, no	0.78	0.69,0.88	< 0.001	0.80	0.71,0.90	< 0.001	0.80	0.71,0.91	< 0.001
Tooth sensitivity, some	1	Ref		1	Ref		1	Ref	
Tooth sensitivity, no	1.10	0.97,1.25	0.130	1.07	0.95,1.22	0.257	1.08	0.96,1.23	0.197
OIDP = 0	1	Ref		1	Ref		1	Ref	
OIDP ≥ 1	1.17	1.04,1.32	0.008	1.13	1,1.27	0.041	1.11	0.99,1.25	0.071
Full health, no				1	Ref		1	Ref	
Full health, yes				0.72	0.65,0.81	< 0.001	0.75	0.67,0.84	< 0.001
Alcohol monthly or never				1	Ref		1	Ref	
Alcohol weekly or more				0.96	0.86,1.08	0.529	1.04	0.93,1.17	0.511
Year of birth – 1932							1	Ref	
Year of birth – 1942							0.95	0.85,1.07	0.443
Unmarried							1	Ref	
Married							0.72	0.64,0.81	< 0.001
*N*	2783			2783			2783		

Model 1: “Oral health”, Model 2: “Oral health + General health” and Model 3: “Oral health + General health + Socio-demographic parameters”

*RR* relative risk, *CI* confidence interval, *p* statistical significance (*p*-value)

**Table 4:** Adjusted logistic regression analysis for men. Dependent variable: seldom/never loneliness vs. always/often loneliness

**Independent variables**	**Model 1**			**Model 2**			**Model 3**		
	**RR**	**95% CI**	**p**	**RR**	**95% CI**	**P**	**RR**	**95% CI**	**p**
Teeth for life, maybe or no	1	Ref		1	Ref		1	Ref	
Teeth for life, yes	0.81	0.67,0.97	0.021	0.84	0.70,1.01	0.057	0.86	0.72,1.04	0.114
Xerostomia day, sometime or no	1	Ref		1	Ref		1	Ref	
Xerostomia day, yes or often	0.96	0.71,1.30	0.798	0.89	0.66,1.20	0.451	0.87	0.64,1.17	0.361
Xerostomia night, sometimes or no	1	Ref		1	Ref		1	Ref	
Xerostomia night, yes or often	1.12	0.91,1.38	0.298	1.07	0.87,1.31	0.544	1.08	0.88,1.33	0.459
Taste Change, some	1	Ref		1	Ref		1	Ref	
Taste change, no	0.70	0.57,0.87	0.001	0.75	0.61,0.93	0.009	0.74	0.60,0.92	0.006
Mouth opening, some	1	Ref		1	Ref		1	Ref	
Mouth Opening, no	0.98	0.77,1.25	0.877	1.03	0.80,1.32	0.830	1.01	0.79,1.30	0.912
Bleeding gum, some	1	Ref		1	Ref		1	Ref	
Bleeding gum, no	0.85	0.70,1.04	0.113	0.85	0.70,1.04	0.118	0.85	0.70,1.03	0.099
Bad breath, some	1	Ref		1	Ref		1	Ref	
Bad breath, no	0.95	0.80,1.14	0.613	0.96	0.81,1.15	0.689	0.97	0.81,1.16	0.744
Tooth sensitivity, some	1	Ref		1	Ref		1	Ref	
Tooth sensitivity, no	1.15	0.96,1.38	0.123	1.13	0.95,1.35	0.172	1.15	0.96,1.37	0.128
OIDP = 0	1	Ref		1	Ref		1	Ref	
OIDP ≥ 1	1.39	1.18,1.65	< 0.001	1.36	1.15,1.61	< 0.001	1.32	1.11,1.56	0.001
Full health, no				1	Ref		1	Ref	
Full health, yes				0.68	0.58,0.80	< 0.001	0.69	0.59,0.81	< 0.001
Alcohol monthly or never				1	Ref		1	Ref	
Alcohol weekly or more				0.97	0.83,1.13	0.672	1.01	0.86,1.18	0.950
Year of birth – 1932							1	Ref	
Year of birth – 1942							0.97	0.79,1.18	0.736
Unmarried							1	Ref	
Married							0.64	0.55,0.75	< 0.001
*N*	2419			2419			2419		

Model 1: “Oral health”, Model 2: “Oral health + General health” and Model 3: “Oral health + General health + Socio-demographic parameters”

*RR* relative risk, *CI* confidence interval, *p* statistical significance (*p*-value)

In the final adjusted model (Model 3), the highest associations related to loneliness were for women (RR = 1.35), being unmarried (RR = 0.69) and self-perceived impaired general health (RR = 0.73). Regarding oral parameters, a lesser belief about keeping their teeth for their remaining life (RR = 0.88), night-time xerostomia (RR = 1.13), taste changes (RR = 0.85), bleeding gums (RR = 0.87), bad breath (RR = 0.86), and impact from OIDP (RR = 1.18) had significant associations with more loneliness (Table 2).

Stratified by sex, being unmarried was significantly associated with more loneliness in both women (RR = 0.72) and men (RR = 0.64) as well as self-perceived impaired general health (RR = 0,75 and RR = 0.64, respectively) (Tables 3 and 4). Among the oral parameters, having night-time xerostomia (RR = 1.14), bleeding gums (RR = 0.88) and bad breath (RR = 0.80) were significantly associated with more loneliness in women (Table 3) while for men taste changes (RR = 0.74) and impact from OIDP (RR = 1.32) (Table 4) were associated with loneliness.

## Discussion

This study shows that multiple socio-demographic and health factors are associated with self-reported loneliness, including several oral health-related variables. For both men and women, 90-year-olds reported greater loneliness than 80-year-olds. Women reported being more affected by loneliness than men and they also had more impaired oral health variables associated with loneliness.

In general terms, the elderly do not constitute a homogenous group of people as their living circumstances are influenced by a number different environmental, social, demographic, economic and health related factors. The very oldest, i.e. 80–90 years old, will increase in the future and according to globally projected figures from the United Nations, the number of persons aged 80 or over is expected to increase threefold from 2017 (137 million) to 2050 (425 million) and close to nine times in 2100 (909 million) [23]. Although some of these very old will remain in a a relatively good physical and mental condition, a great proportion will require extensive physical assistance, cognitive supervision and caregiving. For example, activities of daily living (ADLs) were assessed in people in USA aged 90 to centenarians and ADL difficulty was present in 71% to 97% and ADL dependency in 44% to 92% [24]. All of this may have a direct or indirect effect on perceived loneliness in addition to loneliness being involuntary or voluntary which adds a further complexity to the issue.

The question used to assess loneliness (*“Did you experience more loneliness during the Corona pandemic”*) was related to the period *during* the COVID-19 pandemic. In this regard, studies evaluating loneliness before the pandemic found only a slight increase in loneliness during the pandemic [7, 8]. Therefore, we assume that those who reported “*yes, always*” or “*often* “ more loneliness during the COVID-19 period also experienced loneliness before the outbreak of the pandemic. This assumption has to be viewed with some caution as our study is based on cross-sectional data.

In a Swedish population-based study, targeting the oldest old, viz. 85, 90 and > 95 years, it was found that the prevalence of frequent loneliness was about 49%, 53% and 47% over 3 surveys conducted between 2002–2012 [25]. This was higher than in our samples where 26.9% and 34.3% reported often or always loneliness in the 80- and 90-year-olds, respectively. This is most likely explained by how loneliness was measured in our study but could also be affected by the relatively small sample included in that study [25] and that the previous study was conducted earlier than ours. On the other hand, our figures on loneliness were comparable to those in reviews of elderly people in high income countries (28.5%) and in Swedish elderly people aged over 85 (women 40% and men 30%) [4, 5]. Marital status, women and self-perceived impaired health showed significant positive associations with increased loneliness in our study which is in agreement with other reports [3, 26, 27].

In our study, a relatively large number of self-reported factors reflecting impaired oral health were related to loneliness. Reported perception of not being able to keep their own teeth for their remaining life span, night-time xerostomia, taste changes, bleeding gums, bad breath and impact from OIDP were all related to increased loneliness. Although this cannot directly be translated into clinical findings, it can be deemed probable that loneliness does result in an impaired clinical oral health status. Another interpretation could be that a deteriorated oral status contributes to loneliness consequent to shame or eating problems in social gatherings with other people. In relation to the foregoing, poorer oral health has been reported to even accelerate the risk for social isolation depending on its role in communication [28]. Dental caries and periodontitis, amongst others, often resulting in oral pain or discomfort, are common scenarios in elderly people [29, 30]. These conditions will most likely contribute to increased risk for tooth loss and impaired chewing efficiency which, in turn, may increase the risk for loneliness [12, 31].

Elderly people often exhibit loneliness which may mediate a progressive deterioration in cognitive functions which may affect their ability to communicate their oral discomfort [32, 33]. Based on the findings in this study, 90-year-olds had more loneliness than 80-year-olds, and older women have more such problems than older men. In general, and especially in the case of cognitive impairment, this has to be taken into consideration when planning for dental care and preventive measures in older adults. Dental education should, therefore, focus more extensively on geriatric dentistry including service planning for geriatric oral care and the special needs for oral care in the elderly as has also been suggested by others [34, 35]. In addition, there is a need for integrated care and system-level responses to loneliness among the elderly. Therefore, future research should target geriatric oral health care and its implementation in public polices related to social support programs that counteract loneliness among the elderly.

There are some limitations of the study. The cross-sectional design, self-reported measures, use of a single-item question on loneliness and lack of clinical data limit the validity of the results. Self-selected and involuntary loneliness may have different outcomes on health parameters. Unfortunately, we had no data on whether reported loneliness in our study was self-selected or involuntary, making it impossible to investigate the effect of these two entities. The question that was created by the authors and was intended to evaluate if the participants in the study experienced more loneliness during the Corona pandemic, implicitly meaning compared to before. Although the question could have been constructed with more clarity, we believe that it does give an accurate response as to the level of loneliness compared to before the outbreak of the Corona pandemic. The assumption that those who reported loneliness during the Corona pandemic also experienced loneliness before the pandemic has to be viewed with some caution since it is based on cross-sectional data whereas reports showing such findings in other studies referred to were longitudinal.

## Conclusions

Several oral health-related variables in addition to multiple socio-demographic and health factors were associated with self-reported loneliness. The 90-year-olds reported greater loneliness than the 80-year-olds and women and women were more affected by loneliness than men. In addition, women had a large number of variables related to impaired oral health associated with loneliness compared to men. Considering the global increase in the number of older adults, it is important for both carers of the elderly, and medical and dental care providers to be aware of the commonness of loneliness among the elderly and its negative association with oral health as a case in point.

## Supplementary Information

Supplementary Material 1

Supplementary Material 2

## Data Availability

The data that supports the findings of this study are available from the corresponding author upon reasonable request.
