# Supplementary material for: Loneliness and its associations with oral and general health and socio-demographic factors in 80- and 90-year-old Swedes
Source: BMC Oral Health. 2025 Nov 22;25:1972. doi: 10.1186/s12903-025-07293-4 (PMC12751622; doi:10.1186/s12903-025-07293-4)
Supplement: Supplementary file 1 — Supplementary Material 1 [file 12903_2025_7293_MOESM1_ESM.pdf]

Survey

Oral and dental health of 80 and 90 year olds

Year 2022

Region Östergötland

Region Örebro län

Help us facilitate the processing of your responses

The questionnaire is read by machine. Therefore, when you respond to the survey, please:

- Using a ballpoint pen with black or blue ink
- Write clear numbers, like this: 1 2 3 4 5 6 7 8 9 0
- Mark your answers with a cross, as follows: 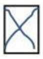
- If you have answered incorrectly, scribble over the entire box with the incorrect X, as follows: 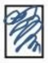

Please contact us

If you have any questions, or if wish to withdraw from the data collection, please call the Survey Factory at 020-12 10 28 or send an email to support@enkatfabriken.se. You can also get chat support at www.enkatfabriken.se/support

Part A. About Your Social Situation

1. Gender

☐ Man ☐ Woman

2. Country of birth

☐ Sweden
☐ Nordic
☐ Other country
☐ Do not know

3. Place of residence

☐ Major urban area ☐ Small urban area ☐ Countryside

4. How many people that you know from before do you meet or converse with during an ordinary week?
(do not count people that you only meet occasionally and who you will not see again)

☐ No ☐ 1-2 ☐ 3-5 ☐ 6-10 ☐ 11-15 ☐ More than 15

5. How do you live?

☐ Own accommodation ☐ Serviced building/social housing ☐ Residential care ☐ Other housing

6. Can you go to a dentist?

☐ Yes ☐ No ☐ Yes, if someone accompanies me ☐ Receives dental care at own accommodation

7. Do you need help in everyday life?

☐ Cleaning
☐ Get cooked food sent home/get food cooked
☐ Shop for food
☐ Personal care
☐ Internet/digital services

8. What education do you have?

☐ Primary school
☐ Higher secondary school, folk high school, 2-year gymnasium, or similar
☐ Gymnasium, 3- or 4-year
☐ University

9. What is your current marital status?

☐ Married/cohabiting
☐ Unmarried
☐ Divorced
☐ Widow(er)

Part B. About your general health

10. Do you consider yourself fully healthy?

- ☐ Yes - absolutely
- ☐ Yes - by and large
- ☐ No - not especially
- ☐ No - absolutely not
- ☐ Has no opinion

11. Do you think that your general health is better or worse than that of your peers?

- ☐ Yes, much better
- ☐ Yes, better
- ☐ About equally good
- ☐ No, worse
- ☐ No, much worse
- ☐ Has no opinion

12. Have you used any prescription medicine in the last 14 days?

☐ Yes ☐ No ☐ Don't remember

13. Have you had contact with a doctor in the last three months?

☐ Yes, several times ☐ Yes, a few times ☐ Yes, once ☐ No ☐ Don't remember

14.

a. How tall are you? ______ cm **b. How much do you weigh?** ______ kg

15. What are your smoking habits?

☐ Smoke daily ☐ Smoke occasionally
☐ Have smoked, but stopped ☐ Never smoked

16. What are your smokeless tobacco habits?

☐ Smokeless tobacco daily ☐ Use smokeless tobacco occasionally
☐ Used smokeless tobacco before, but stopped ☐ Never used smokeless tobacco

17. How often do you drink strong beer, wine or spirits?

☐ More than a couple of times/week
☐ Approximately a couple of times/week
☐ Approximately once/week
☐ Approximately a couple of times/month
☐ Never

Part C. About Your Mouth and Your Teeth

18. Do you think you can keep your teeth for life?

☐ Yes, absolutely
☐ Yes maybe
☐ I don't know
☐ No, probably not
☐ No, absolutely not

19. Are you generally satisfied with your teeth?

- ☐ Yes, very satisfied
- ☐ Yes, by and large satisfied
- ☐ No, not especially satisfied
- ☐ No, absolutely not satisfied

20. Can you chew all kinds of food?

- ☐ Very good
- ☐ Rather good
- ☐ Not so good
- ☐ Bad

21. Are you satisfied with the appearance of your teeth?

- ☐ Yes, very pleased
- ☐ Yes, by and large pleased
- ☐ No, not especially pleased
- ☐ No, absolutely not pleased

22. Does your mouth feel dry?

Daytime:
- ☐ Yes, often
- ☐ Yes, sometimes
- ☐ No, seldom
- ☐ No, never

Night-time:
- ☐ Yes, often
- ☐ Yes, sometimes
- ☐ No, seldom
- ☐ No, never

23. When was the last time you had a toothache?

- ☐ In the last three months
- ☐ In the last year
- ☐ More than a year ago
- ☐ Never had toothache
- ☐ Don't remember

24. Which oral care product(s) are you using?

Specify how often you use each agent.

	Rarely/ never	Once every week	Once a day	Twice a day	More than twice a day
Toothbrush	☐	☐	☐	☐	☐
Toothpaste with fluoride	☐	☐	☐	☐	☐
Tooth sticks / gap brush	☐	☐	☐	☐	☐
Floss	☐	☐	☐	☐	☐
Fluoride tablets	☐	☐	☐	☐	☐
Fluoride irrigation	☐	☐	☐	☐	☐

25. How many of your own teeth do you have?

- ☐ All teeth left
- ☐ Almost no teeth left
- ☐ Missing a single tooth
- ☐ Edentulous
- ☐ Missing rather many teeth

26. Have you had any teeth removed in the last 12 months?

☐ No ☐ Yes, one tooth ☐ Yes, some teeth ☐ Yes, many teeth

27. You can have many different problems from the mouth and teeth. Do you feel that you have any of the following problems or troubles? Think it through and answer every example.

	No trouble	Some trouble	Rather much trouble	Great trouble
Teeth color	☐	☐	☐	☐
Teeth shape	☐	☐	☐	☐
Teeth are not straight	☐	☐	☐	☐
Over- or underbite	☐	☐	☐	☐
Too much space between teeth	☐	☐	☐	☐
Too tight spaces between teeth	☐	☐	☐	☐
Burning mouth	☐	☐	☐	☐
Oral wounds or blisters	☐	☐	☐	☐
Taste changes	☐	☐	☐	☐
Jaw joint pain	☐	☐	☐	☐
Clicking or grating from jaw joint	☐	☐	☐	☐
Mouth cannot open wide	☐	☐	☐	☐
Tooth grinding/clenching	☐	☐	☐	☐
Bleeding gums	☐	☐	☐	☐
Bad breath	☐	☐	☐	☐
Discomfort from dental fillings	☐	☐	☐	☐
Tooth sensitivity	☐	☐	☐	☐
Tooth wear	☐	☐	☐	☐

28. You can have different attitudes to your teeth. Here are some statements and opinions that may occur. Please indicate your position on them:

	Agree, absolutely	Agree, by and large	Don't agree	Absolutely don't agree
"Having beautiful and perfect teeth is very important for how you are treated by other people"	☐	☐	☐	☐
"Minor beauty defects on the teeth are not important, as long as they work"	☐	☐	☐	☐
"If missing teeth are visible it is something to be ashamed of"	☐	☐	☐	☐
"It doesn't matter how you look in your mouth, as long as you can chew the food you like"	☐	☐	☐	☐

29. Are you generally satisfied with Your mouth and Your teeth?

Yes, very satisfied	Yes, by and large satisfied	No, not especially satisfied	No, absolutely not satisfied
☐	☐	☐	☐

Part D. About your dental care

30. Where have you mainly received dental care **over** the past five years?

- ☐ Private
- ☐ Public Dental Health Service
- ☐ No dental care
- ☐ Other, namely:

31. Approximately how often do you go to dental care?

☐ Two or more times/year ☐ Once per year ☐ Every two years ☐ More seldom

32. Have you been forced to forgo dental appointments in the past year because you could not afford the appointment?

☐ Yes - multiple times ☐ Yes, occasionally ☐ No

33. In recent years, have you had to forgo the dental treatment suggested by your dentist because you could not afford it?

☐ Yes ☐ No

34. Have you changed your visiting habits with dentistry in recent years?

☐ Yes, I go more often ☐ Yes, I go more seldom ☐ No ☐ Don't know

35. Approximately how much have you paid for your dental care yourself in the last year?

☐ Nothing ☐ 1-2 000 SEK ☐ 2 001-8 000 SEK ☐ More than 8 000 SEK ☐ Don't remember

36. Have you visited a dental hygienist in the past year?

☐ Yes ☐ No ☐ Don't know

37. You can have many different materials and designs in fillings and tooth replacements.
What fillings/replacements look like in your mouth? Specify all the options that apply to You! Do you have:

- ☐ "White" fillings
- ☐ Porcelain
- ☐ Gold
- ☐ Implant
- ☐ Amalgam
- ☐ Provisional fillings
- ☐ Removable partial denture upper jaw
- ☐ Removable partial denture lower jaw
- ☐ Complete denture upper jaw
- ☐ Complete denture lower jaw

38. Do you have pain in your temples, face, jaw joints or jaws once a week or more often?

☐ Yes ☐ No

39. Does it hurt when you open your mouth or chew once a week or more often?

☐ Yes ☐ No

40. Do you have jaw locking once a week or more often?

☐ Yes ☐ No

41. Have you had problems with dental caries (holes in the teeth) in the past year?

☐ Yes ☐ No ☐ Don't know

42. Have you had problems with periodontitis (gum disease)?

☐ Yes ☐ No ☐ Don't know

43. Are you generally satisfied or dissatisfied with the dental care you have received in the past?

☐ Very satisfied ☐ By and large satisfied ☐ Rather dissatisfied ☐ Very dissatisfied

44. Have you generally had the opportunity to go to the dentist you wanted to be treated by?

☐ Yes, always ☐ Yes, mostly ☐ Only occasionally ☐ No, seldom ☐ No, never

45. Do you think it is important to be able to go to the same dentist/dental hygienist every time?

☐ Yes, very important ☐ Yes, rather important ☐ No, not so important ☐ No, not important at all

46. Do you regularly go to your current dentist?

☐ Yes ☐ No

47. Have you ever wanted to change dentist in the last five years because you have been dissatisfied?

☐ Yes, several times ☐ Yes, occasionally ☐ No ☐ Don't remember

48. Have you ever changed your dentist in the last five years because you have been dissatisfied?

☐ Yes, several times ☐ Yes, occasionally ☐ No ☐ Don't remember

49. Did you have any really unpleasant or frightening experience of dental care during your childhood or adolescence (up to about 20 years old)

☐ Yes, several times ☐ Yes, occasionally ☐ No ☐ Don't remember

Part E. About your latest visit to dental care

50. Where was your last dental visit?

☐ Public Dental Health ☐ private ☐ Other ☐ Don't remember

51. When was your last dental visit?

☐ Less than 1 year ago ☐ 1 - 3 years ago ☐ 3 - 5 years ago ☐ More than 5 years

52. Who initiated your last dental visit?

☐ You or your family members, e.g. emergency visits, new appointment
☐ Dental care, e.g. regular recall
☐ Don't remember

53. We want to know how you experienced your <u>most recent</u> dental visit in various respects.
Put a check in the box that best matches how you felt during your visit

No pain	☐	☐	☐	☐	☐	Unbearable pain
No discomfort	☐	☐	☐	☐	☐	Very uncomfortable
No worry	☐	☐	☐	☐	☐	Much worry
Good care	☐	☐	☐	☐	☐	Poor care

54. During any of your most recent dental visits, did you receive information about any or more of the following?	Yes	No	Don't know
Oral hygiene	☐	☐	☐
Diet	☐	☐	☐
Fluoride	☐	☐	☐
Tobacco	☐	☐	☐
Cost for treatment	☐	☐	☐

Part F. About your dental health

55. How often have you had problems with teeth or dentures during the last six months that made it difficult for you...	Daily or almost daily	Once or twice a week	1-2 times a month	Less than 1 time a month	Never
to eat and enjoy the food?	☐	☐	☐	☐	☐
with speech and pronunciation?	☐	☐	☐	☐	☐
taking care of your oral hygiene?	☐	☐	☐	☐	☐
sleeping or relaxing?	☐	☐	☐	☐	☐
to smile, laugh or show your teeth without being embarrassed?	☐	☐	☐	☐	☐
to maintain emotional stability without getting irritated?	☐	☐	☐	☐	☐
to enjoy interaction with other people?	☐	☐	☐	☐	☐
to do your daily activities?	☐	☐	☐	☐	☐

Part G. About the time of the Coronavirus pandemic

56. Have you visited dental care during the Coronavirus pandemic? *Check the options that are right for you.*

☐ Yes, for planned examination/treatment ☐ Yes, for emergency treatment ☐ No

57. Have you had to postpone scheduled dental care during the Coronavirus pandemic? *Tick the appropriate option for you.*

☐ Yes, I wanted to/couldn’t go ☐ Yes, the dentist did not accept ☐ No

58. During the Coronavirus pandemic	Yes, always	Often	Seldom	Never
Did you experience more loneliness during the Corona pandemic?	☐	☐	☐	☐
Did you feel very worried about getting ill?	☐	☐	☐	☐
Lost contact with your friends and relatives??	☐	☐	☐	☐
Did you have problems with digital communication in your contacts with healthcare/dentistry?	☐	☐	☐	☐

59. Have you been ill or tested positive for Corona/Covid-19?

☐ Yes ☐ No ☐ Don't know

If yes, do you allow us to return with a follow-up questionnaire about Corona/Covid-19??

☐ Yes ☐ No

Part H. Other comments

Thank you for taking the time to respond!
