# Supplementary material for: Loneliness and its associations with oral and general health and socio-demographic factors in 80- and 90-year-old Swedes
Source: BMC Oral Health. 2025 Nov 22;25:1972. doi: 10.1186/s12903-025-07293-4 (PMC12751622; doi:10.1186/s12903-025-07293-4)
Supplement: Supplementary file 2 — Supplementary Material 2 [file 12903_2025_7293_MOESM2_ESM.docx]

Table S1: Number of missing for the variables included in the regression analyses.

| **Independent variables** | **Missing** |
| --- | --- |
| Teeth for life | 83 |
| Xerostomia day | 333 |
| Xerostomia night | 605 |
| Taste Change | 308 |
| Mouth opening | 248 |
| Bleeding gum | 288 |
| Bad breath | 299 |
| Tooth sensitivity | 455 |
| OIDP | 141 |
| Full health | 147 |
| Alcohol | 80 |
| Year of birth | 0 |
| Marital status | 73 |
| Gender | 0 |
| N missing (imputed) | *1684* |
| N complete | *3518* |
| N total | *5202* |
